# Individualized estimates of intensity-modulated radiotherapy plans after breast conservation surgery for left-sided breast cancer

**DOI:** 10.1186/s12957-023-02936-8

**Published:** 2023-02-23

**Authors:** Yong Wang, Lingqin Ni, Shenpeng Ying, Yuanyuan Xu, Weijun Chen, Yanmei Liu

**Affiliations:** Department of Radiotherapy, Taizhou Central Hospital (Taizhou University Hospital), No.999 Donghai Road, Taizhou, 318000 Zhejiang People’s Republic of China

**Keywords:** Breast cancer, Radiation therapy, Cardiothoracic ratio, Lung center distance, Maximum distance of the heart

## Abstract

**Objectives:**

The purpose of this study was to explore the influence of individual patient factors, such as volume of the planning target volume (PTV) (V_PTV_), cardiothoracic ratio (CTR), central lung distance (CLD), and maximal heart distance (MHD), on the design of treatment plans in terms of target dose coverage, integral dose, and dose to organs at risk (OAR) in early breast cancer.

**Methods:**

Ninety-six patients were selected for this study. Radiation doses of 50 Gy and a simultaneous dose of 60 Gy in 25 fractions were administered to the whole breast and tumor bed, respectively. The intensity modulation plan (IMRT) of each patient uses both physical parameters and an equivalent uniform dose (EUD) to optimize the target function. Univariate and multivariate linear regression were used to analyze the relationship between predictive impact factors and OAR percent dose volume, conformity index (CI), and homogeneity index (HI).

**Results:**

The average CI and HI values of the left breast cancer plan were 0.595 ± 0.071 (0.3–0.72) and 1.095 ± 0.023 (1.06–1.18), respectively. The CTR (*B* = 0.21, *P* = 0.045), V_PTV_ (*B* = 0.63, *P* = 0.000), volume of the lung (V_lung_) (*B* =  − 0.29, *P* = 0.005), and MHD (*B* = 0.22, *P* = 0.041) were identified as factors influencing the CI index of the left breast cancer intensity modulation plan. V_PTV_ (*B* = 1.087, *P* = 0.022) was identified as the influencing factor of the *HI* index of the left breast cancer intensity modulation plan. volume of the heart (V_heart_) (*B* =  − 0.43, *P* = 0.001) and CLD (*B* = 0.28, *P* = 0.008) were influencing factors of the volume of lung (V_lung20_) of the lung. The prediction formulas for left-sided breast cancer are noted as follows: CI = 0.459 + 0.19CTR-0.16CLD, V_lung_10 = 35.5–0.02V_heart_; and V_lung_20 = 21.48 + 2.8CLD-0.018V_heart_.

**Conclusions:**

CTR, CLD, and MHD can predict the rationality of the parameters of the left breast cancer IMRT. The calculation formula generated based on this information can help the physicist choose the optimal radiation field setting method and improve the quality of the treatment plan.

## Introduction

Breast cancer is a malignant tumor that threatens the lives and health of women worldwide. Regarding the postponement of marriage and childbirth, the incidence of breast cancer tends to increase. At present, approximately 304,000 new breast cancer cases are diagnosed in our country every year. With the improvement of breast cancer diagnosis and treatment, the 5-year survival rate has reached 83.2% [1]. Given the improvement of medical standards, the early detection rate of breast cancer has further increased, and the breast-conserving rate of breast cancer patients has also further increased [2]. Therefore, postoperative adjuvant whole breast radiation therapy (WBRT) is an important part of the current standard treatment for early breast cancer, which can bring long-term local control and survival [3, 4].

The side effects of long-term radiotherapy are a problem that cannot be ignored with the prolonged survival of breast cancer patients. Given the special anatomical relationship between the breast and heart, long-term heart damage caused by radiotherapy has also received increasing attention [5]. RP is one of the most common and poor prognostic complications of postoperative radiotherapy for breast cancer. Previous studies have confirmed that radiotherapy doses, lung exposure, and other indicators are high-risk factors for radiation pneumonitis [6]. At present, inverse intensity-modulated radiotherapy technology has been widely used in whole breast radiotherapy and is able to deliver conformal and tumoricidal doses to the target [7]; thus, the target area has a higher coverage rate and less OAR exposure. However, the design of the IMRT plan is affected not only by the angle of the field and the multileaf grating but also by the patient’s own factors. Vivekanandan [8] performed a more detailed study on the design of the static intensity modulation plan from the angle of the frame and the multileaf collimator and applied it in script mode. Although the efficiency of the plan is improved, the patient’s target area and the anatomical spatial structure of the OAR occasionally need to be re-established and optimized, which wastes considerable time and energy and reduces the efficiency of work.

This study adopts the commonly used static inverse intensity modulation plan and expects to generate the corresponding formula through the analysis of the dose volume of V_PTV_, CTR, CLD, MHD and CI, HI, OAR, and the bivariate correlation analysis with OAR. This can predict whether the intensity modulation plan for breast cancer patients can meet clinical requirements for physicists.

## Methods and materials

### Patients

Ninety-six patients were selected for this study. All specimens were released by the patients upon signing informed consent forms. Thea analysis of human material was approved by the local ethics committee.

### Computed tomography (CT) images

The positioning technician instructed the patients to lie in a supine position with their arms raised above their head and to hold the arm support. After 5 min of free breathing, the patient was marked with a cross line according to the position of the laser line, and a CT scan was performed with a thickness of 5 mm. The scan range was from the upper edge of the throat to 10 cm below the breast fold. The image is transmitted to Pinnacle Treatment Planning System (TPS).

### Contouring

All contours were performed by clinicians using Eclipse (version 15.5) according to the Radiation Therapy Oncology Group (RTOG) Breast Cancer Atlas for Radiation Therapy Planning: Consensus Definitions [9].

The clinical target volume (CTV) consisted of the breast glands and the lymphatic drainage area of the chest wall under the breast. Levels II–III of the axial and supraclavicular lymph node areas are needed if the patient has axillary lymph node metastasis. The tumor bed was also delineated by preoperative physical examination, surgical marks, and surgical wounds. The PTV was obtained by expanding the CTV by 7 mm, but the front boundary was 5 mm below the skin surface. The spinal cord, ipsilateral lung, contralateral lung, contralateral breast, heart, and left anterior descending artery were delineated as OARs.

The HI and CI formulas of PTV [10] are noted below:

1 $$\mathrm{HI}\;=\;{\mathrm D}_{5\%}/{\mathrm D}_{95\%}$$

2 $$\mathrm{CI}\:=\:{\mathrm{PTV}}_{95\%}/\mathrm{PTV}\:\times\:{\mathrm{PTV}}_{95\%}/{\mathrm V}_{95\%}$$

In Formula (1), D_5__%_ is the dose received by 5% of the PTV, and D_95%_ is the dose received by the volume of 95% of the PTV. In Formula (2), PTV_95%_ is 95% of PTV, and V_95%_ indicates the volume included in the 95% isodose line. The closer the values of CI and HI are to 1, the better the conformity and uniformity of the dose distribution.

### Prescriptions and planning

**Fig. 1:**
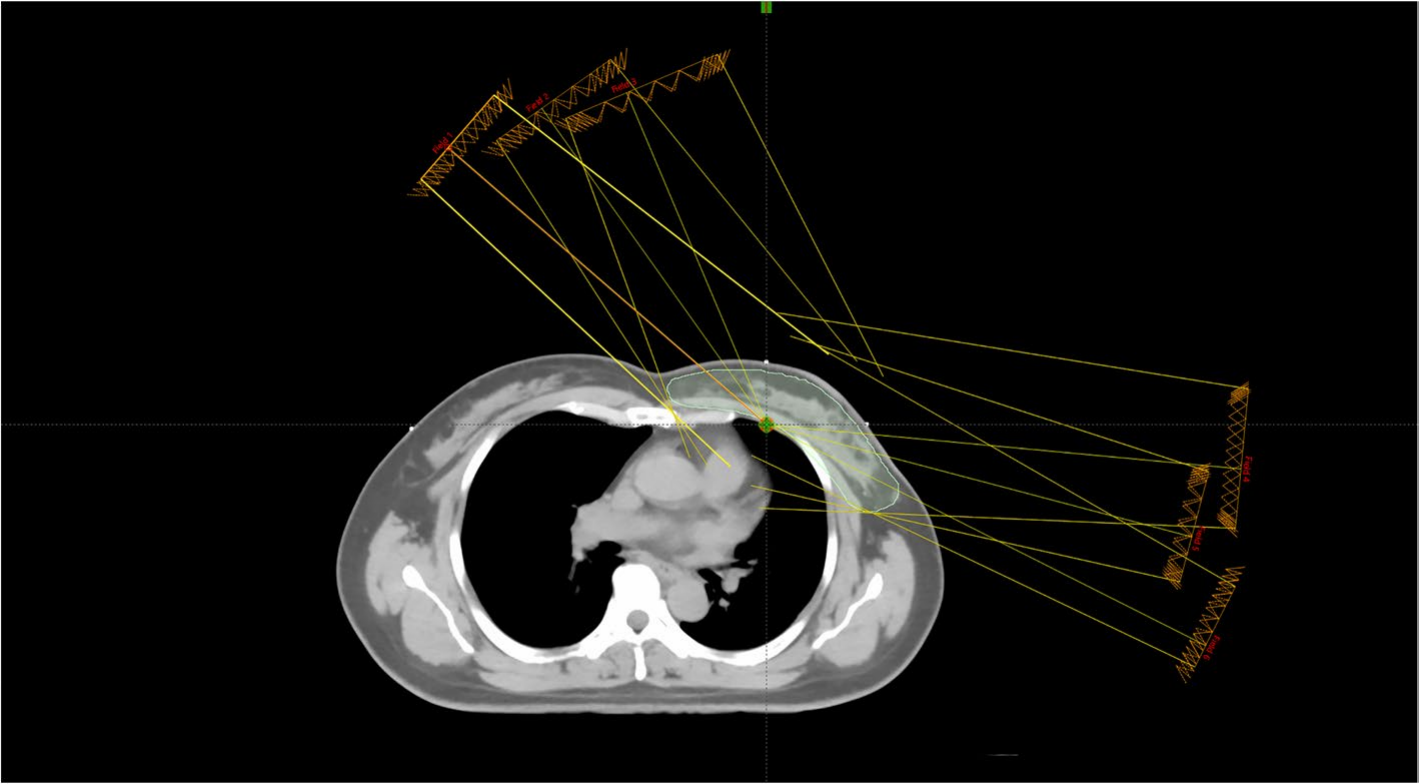
Representative of left breast intensity-modulated radiotherapy program

Radiation doses of 50 Gy and a simultaneous dose of 60 Gy in 25 fractions were administered to the whole breast and tumor bed, respectively. The three paired tangential beams were adopted, which had an average of 6 ~ 8 subfields (Fig. 1). The minimum subfield area must be > 10 cm^2^, and the minimum subfield jump number must be > 10 MU. A 10–15° difference is between the gantry angles in the same direction. The physical dose limit and EUD were both applied to optimize the objective function.

### Measurement of predictive factors and evaluation of indicators

#### CTR

**Fig. 2:**
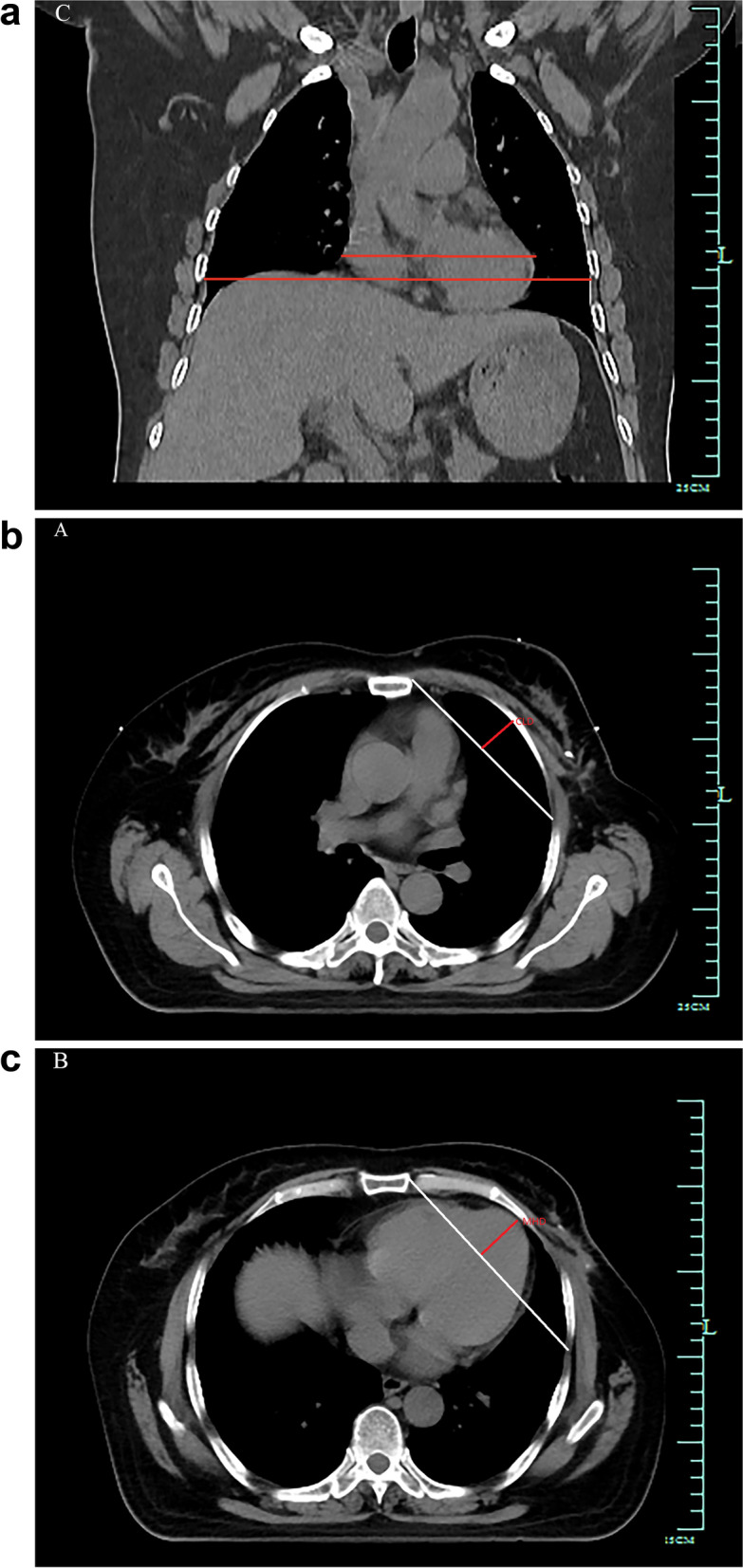
**a** Schematic diagram of measurement of cardiothoracic ratio in breast cancer patients. **b** Schematic diagram of the maximum vertical distance of the lungs of breast cancer patients. **c** Schematic diagram of the maximum vertical distance to the heart of breast cancer patients

The ratio of the maximum transverse diameter of the heart measured on the CT coronal image and the distance between the inner wall of the thorax measured across the top of the diaphragm (Fig. 2a).

#### CLD

A vertical line is drawn based on the layer closest to the midline of the target area of the inscribed field, and a horizontal line is made on the layer on the backmost side of the target area of the outer incisal field on the CT cross-sectional image. To move to the same level to construct a right-angled triangle by system software, a vertical line was drawn on the hypotenuse to the inside of the chest wall, and the vertical line with the longest distance was selected (Fig. 2b).

#### MHD

To draw a straight line connecting the inner boundary of the incisor field and the inner boundary of the outer incisor field on the CT cross-section where the apex is located, which is the maximum distance from the top of the apex to the line (Fig. 2c).

#### Treatment plan evaluation

The dosimetric study of the treatment plan was based on the following parameters extracted from the dose-volume histogram (DVH): HI; CI; V_PTV_; heart V30, V10, V5, and D_max_ and MHD, the D_mean_ of the left anterior descending (LAD), and the V20, V10, V5, and D_mean_ of ipsilateral lungs.

### Statistical analysis

The correlation was determined through the univariate linear regression analysis, and then, the multiple linear regression analysis is carried out. The index with a *p* value < 0.05 was selected, and the corresponding prediction formula was generated according to the coefficient of the index. All statistical analyses were performed using SPSS 22 software.

## Results

### The impacts on CI and HI

The average CI and HI values of the Breast Cancer Program were CI = 0.595 ± 0.071 (0.3—0.72).

Univariate linear regression showed that CTR (*B* = 0.21, *P* = 0.045), V_PTV_ (*B* = 0.63, *P* = 0.000), V_lung_ (*B* =  − 0.29, *P* = 0.005), CLD (*B* =  − 0.21, *P* = *0.047*), and MHD (*B* = 0.22, *P* = 0.041) were all influencing factors of the CI index of the left breast cancer intensity modulation plan. However, multivariate linear regression showed that CLD (*B* =  − 0.16, *P* = 0.048), CTR (*B* = 0.19, *P* = 0.015), and V_PTV_ (*B* = 0.00, *P* = 0.000) were independent influencing factors of the left CI (Table 1).

**Table 1:** Univariate and multiple linear regression analysis of CI

Variables	Structures	*CI*			
		*R*^*2*^	95%CI	*B*	*P*
Unary linear regression					
	CLD	0.044	− 0.06–0	− 0.21	0.047
	CTR	0.045	0.007–0.607	0.21	0.045
	V_PTV_	0.39	0–0	0.63	0.000
	MHD	0.047	0.001–0.051	0.22	0.041
	V_lung_	0.085	0–0	− 0.29	0.005
Multiple linear regression					
	CLD	0.459	− 0.046–0	− 0.16	0.048
	CTR	0.459	0.057–0.515	0.19	0.015
	V_PTV_	0.459	0–0	0	0.000

Both univariate and multivariate linear regression showed that V_PTV_ (*B* = 1.087, *P* = 0.022) was a factor influencing the *HI* index of the left breast cancer intensity modulation plan, but CLD (*B* = 0.001, *P* = *0.224*) was not (Table 2).

**Table 2:** Univariate and multiple linear regression analysis of HI

Variables	Structures	*HI*			
		*R*^*2*^	95%*CI*	*B*	*P*
Unary linear regression					
	CLD	0.017	− 0.001–0.003	0.001	0.224
	CTR	0.027	− 0.172–0.02	− 0.076	0.119
	V_PTV_	0.058	0–0	1.087	0.022
	MHD	0.007	− 0.004–0.01	0.003	0.440
Multiple linear regression					
	V_PTV_	0.058	0–0	1.087	0.022

### The relationship between OAR volume and CLD, MHD, and OAR percent dose

For the dosimetry studies of the lung, we analyzed some common factors, such as V_lung5_, V_lung10_, V_lung20_, and D_meanlung,_ which are related to radiation pneumonitis. The univariate linear regression showed that V_lung_ (*B* = 0.29, *P* = 0.006), V_PTV_ (*B* = 0.22, *P* = *0.037*), V_heart_ (*B* =  − 0.43, *P* = 0.001), CTR (*B* =  − 0.3, *P* = 0.005), and CLD (*B* = 0.28, *P* = 0.008) were the influencing factors of V_lung20_. However, only V_heart_ (*B* =  − 0.41, *P* = 0.001) and CLD (*B* = 0.28, *P* = 0.018) achieved significant differences in the multiple linear regression (Table 3).

**Table 3:** Unary and multiple linear regression analysis of the relationship between predictive factors MHD, CTR, V_PTV_, CLD, and lung dose volume

Variables	Structures	Lungs					
		V_20_		V_10_		Dmean	
		Unary linear regression	Multiple linear regression	Unary linear regression	Multiple linear regression	Unary linear regression	Multiple linear regression
V_lung_	*R*^2^	0.08		0.09		0.05	0.05
	95%CI	− 0.002–0.009		0–0.01		0.04–1.74	0.04–1.74
	*P*	0.006		0.003		0.040	0.040
	B	0.29		0.31		0.22	0.22
V_PTV_	*R*^2^	0.05		0.05			
	95%CI	0–0.01		0–0.01			
	*P*	0.037		0.032			
	B	0.22		0.23			
V_heart_	*R*^2^	0.18	0.26	0.19	0.19		
	95%CI	− 0.03–0.009	− 0.03–0.01	− 0.03–0.01	− 0.03–0.01		
	*P*	0.001	0.001	0.001	0.001		
	B	− 0.43	− 0.41	− 0.44	− 0.44		
CTR	*R*^2^	0.09		0.08			
	95%CI	− 43.51–8.12		51.83—-9.56			
	*P*	0.005		0.005			
	B	− 0.3		− 0.29			
CLD	*R*^2^	0.08	0.26				
	95%CI	0.67–4.26	0.5–5.1				
	*P*	0.008	0.018				
	B	0.28	0.28				

In addition, we also explored the impact of the above variables on cardiac-related monitoring indicators. Regarding the V_30_ of the heart, single-factor analysis suggests that MHD (*B* = 0.48, *P* = 0.000), CTR (*B* = 0.436, *P* = 0.000), and V_lung_ (*B* =  − 0.52, *P* = 0.000) were the influencing factors. Multifactor analysis suggested that MHD (*B* = 0.37, *P* = 0.000) and V_lung_ (*B* =  − 0.42, *P* = 0.000) were influencing factors, whereas CTR was not. Regarding the Dmean of the heart, the multivariate analysis also obtained similar results with V_30_. Both MHD (*B* = 0.28, *P* = 0.005) and V_lung_ (*B* =  − 0.33, *P* = 0.001) were influencing factors (Table 4).

**Table 4:** Unary and multiple linear regression analysis of the relationship between predictive factors MHD, CTR, V_PTV,_ CLD and heart, and LAD dose volume

Variables	Structures			Heart					LAD	
		V_30_		Dmax		Dmean			Dmean	
		Unary linear regression	Multiple linear regression	Unary linear regression	Multiple linear regression	Unary linear regression		Multiple linear regression	Unary linear regression	Multiple linear regression
MHD	*R*^2^	0.24	0.4	0.16	0.16	0.14		0.24	0.13	
	95%CI	1.87–4.19	1.23–3.39	140.25–408.38	140.25–408.38	65.4–214.5		32.43–179.09	2428.65–8326.54	
	*P*	0.000	0.000	0.000	0.000	0.000		0.005	0.000	
	B	0.48	0.37	0.4	0.4	0.37		0.28	0.36	
CTR	*R*^2^	0.19		0.05		0.06				
	95%CI	18.5–47.30		144.5–3581		164.89–2043.73				
	*P*	0.000		0.034		0.022				
	B	0.436		0.22		0.24				
V_lung_	*R*^2^	0.27	0.4	0.08		0.16		0.24	0.14	0.21
	95%CI	− 0.01–0.005	− 0.01–0.004	− 0.88–0.16		− 0.58–0.21		− 0.51–0.13	− 1.81–0.55	− 2.41–0.79
	*P*	0.000	0.000	0.006		0.000		0.001	0.000	0.000
	B	− 0.52	− 0.42	− 0.29		− 0.41		− 0.33	− 0.37	− 0.46
V_PTV_	*R*^2^					0.06			0.13	
	95%CI					0.03–0.35			− 1.42–0.41	
	*P*					0.020			0.001	
	B					0.24			-0.36	
V_heart_	*R*^2^								0.09	
	95%CI								0.37—3.89	
	*P*								0.019	
	B								0.31	

In addition, we also performed a variable analysis on LAD. Single-factor analysis suggested that the Dmean of LAD was affected by MHD (*B* = 0.36, *P* = 0.000), V_lung_ (*B* =  − 0.37, *P* = 0.000), V_PTV_ (*B* =  − 0.36, *P* = 0.001), and V_heart_ (*B* = 0.31, *P* = 0.019), whereas multivariate analysis only suggests that V_lung_ (*B* = 0.31, *P* = 0.019) is the influencing factor of the Dmean (Table 4).

### The prediction formula

$$\begin{array}{l}\mathrm{HI}:\;\mathrm{consant}\left(1.087\right)\\\mathrm{CI}:\;0.459\;+\;0.19\mathrm{CTR}\;-\;0.16\mathrm{CLD}\end{array}$$

### Lung

$$\begin{array}{l}{\mathrm D}_{\mathrm{mean}}:207.5\hspace{0.17em}+\hspace{0.17em}0.89{\mathrm V}_{\mathrm{lung}}\\{\mathrm V}_{\mathrm{lung}}10:35.5\hspace{0.17em}-\hspace{0.17em}0.02{\mathrm V}_{\mathrm{heart}}\\{\mathrm V}_{\mathrm{lung}}20:21.48\hspace{0.17em}+\hspace{0.17em}2.8\mathrm{CLD}-0.018{\mathrm V}_{\mathrm{heart}}\end{array}$$

### Heart

$$\begin{array}{l}{\mathrm{LAD}}_{\mathrm{mean}}=3829-1.59{\mathrm V}_{\mathrm{lung}}\\{\mathrm D}_{\mathrm{mean}}=889.56+105.76\mathrm{MHD}-0.32{\mathrm V}_{\mathrm{lung}}\\{\mathrm D}_{\mathrm{max}}=4699+274.31\mathrm{MHD}\\{\mathrm V}_{\mathrm{heart}}30=9.4+2.3\mathrm{MHD}-0.007{\mathrm V}_{\mathrm{lung}}\end{array}$$

## Discussion

Adjuvant radiotherapy after breast-conserving breast cancer surgery can effectively improve the local control rate [10], reduce the risk of tumor recurrence [11], and prolong patient survival [12]. However, with the prolonged survival period, the long-term adverse reactions caused by radiotherapy are obvious. How to reduce the incidence of adverse reactions by optimizing radiotherapy techniques and methods is a current research hotspot. The CI, HI value, and OAR dose volume of the intensity modulation plan are important indicators for the evaluation of the treatment plan. In the course of clinical practice, the optimal dose is not reached, and the indicators are compromised. The CI and HI values of the intensity modulation plan are affected not only by the angle of the gantry and MLC but also by the OAR dose limitation and spatial distance. The increase in the number of OARs and the limitation of dose volume complicate planning optimization. To reduce the repeated modification and optimization of parameters by physicists and improve the efficiency of planning design, this study uses PTV and OAR as the objective functions to explore patient factors that affect plan design.

To ensure the reliability of the research results, studies should try to exclude the influence of nonpatient factors on the research results. CI is affected by the number of firing fields, the number of subfields and the angle of the gantry. Previous studies [13] on radiotherapy dosimetry of different intensity modulation methods after breast-conserving surgery showed that nonpatient factors, such as increasing the number of subfields, vertical incidence, and other methods, can increase the CI value of the intensity modulation plan but simultaneously increase the OAR low-dose exposure volume. Therefore, all patients in this study used full tangent field irradiation with the same gantry conditions, number of fields, and number of subfields. The heart and lungs are important OARs in the design of radiotherapy after breast-conserving breast cancer surgery. The indicators related to the heart and lungs are important factors influencing the design of the plan, and previous studies have not conducted further studies on these OARs. This study enrolled 96 patients with left-side breast cancer after breast-conserving radiotherapy and explored the influence of heart- and lung-related patient factors, such as CLD, CTR, MHD, and V_PTV_, on the design of radiotherapy plans.

The CI and HI values are important indicators of the intensity modulation plan. The average CI and HI values of the intensity modulation plan for left-side breast cancer patients in the supine position were similar to those previously reported in the literature [14]. In this study, the relationship between the predictive impact factors of breast cancer IMRT and the CI and HI values identified through univariate linear regression analysis showed that V_PTV_, MHD, and CTR were the influencing factors of the CI value of left-side breast cancer. Multiple linear regression analysis showed that CTR, CLD, and V_PTV_ were independent influencing factors of CI, and measuring the CTR value of patients can quickly predict the CI value of patients with left breast cancer. Both univariate linear regression and multiple linear regression show that V_PTV_ is the only factor impacting HI. HI was mainly affected by the patient’s own breast volume, which differs from the results regarding CI. From another aspect, this finding also verifies the importance of individualized treatment plans.

Cardiovascular disease is the number one cause of death in elderly breast cancer survivors. Studies [15] have found that left breast radiotherapy can cause damage to the entire length of the left coronary artery, including the proximal and middle and distal ends. Given the increasing number of breast cancer survivors with a history of breast radiotherapy, more attention should be given to these patients in clinical practice. The risk of severe coronary stenosis may occur during the period. When formulating a radiotherapy plan, how to control the radiation dose to the heart is also difficult to address and serves as the focus of the plan. The heart is an important dose-limiting organ in the treatment of left-side breast cancer with radiotherapy. The characteristics of the heart as a parallel organ determine that the radiation damage of the heart is related to its radiation dose or volume [16] (especially V_heart_ 30, D_max_heart). The univariate linear regression analysis of this study found that MHD, CTR, and V_lung_ were the influencing factors of cardiac V_heart_ 30, and the results of multivariate linear regression analysis showed that MHD and V_lung_ were independent influencing factors of left breast cancer V_heart_ 30_._ The prediction formula was V_heart_ 30 = 9.4 + 2.3MHD-0.007 V_lung_. De Almeida [17] used ECG gating technology to control the volume of apical tissue in breast cancer IMRT to demonstrate that MHD can predict the amount of cardiac tissue, and their methodology is similar to ours.

The CTR of 96 female breast cancer patients in this group was 0.51 ± 0.05, which was consistent with the adult CTR [14]. This study performed linear regression analysis on the predictors and dose volume of OARs. Univariate linear regression analysis showed that MHD, CTR, and V_lung_ were influencing factors of D_maxHeart_. Multiple linear regression showed that MHD was an independent influencing factor of D_maxHeart_. MHD and D_maxHeart_ were positively correlated. The prediction formula was D_maxHeart_ = 4699 + 274.31 MHD, which represents the first use of MHD in a prediction formula. Chest radiotherapy has an important influence on the cause of heart-related diseases [18, 19]. Another study [15] found that left breast radiotherapy can cause damage to the entire length of the left coronary artery, including the proximal and middle and distal ends, and LAD is one of the main monitoring indicators. The univariate regression equation in this study suggested that MHD is the impact factor of the Dmean of the LAD, whereas the multiple regression equation suggests that only V_PTV_ is its impact factor. Vees et al. [9] studied left breast tangent field irradiation of the heart, and the results showed that the V_heart_ 30 of the heart is affected by V_PTV_. The results of the univariate linear regression analysis of this study showed that the heart volume of patients with left-side breast cancer and the dose volume of each gradient were not correlated, and the results suggest that V_PTV_ cannot predict the value of each parameter of the heart in the reverse intensity modulation plan.

RP is one of the main complications that affect the prognosis and quality of life of patients [20]. Effective assessment and management of RP can improve the quality of life of patients [21]. Studies have reported that the incidence of RP for breast cancer patients receiving intensity-modulated radiotherapy is 10.6% [22]. Previous literature has shown that V_lung_ 20, V_lung_ 10, and V_lung_ 5 are predictors of RP, and controlling for V_lung_ 20, V_lung_ 10, and V_lung_ 5 is effective in reducing the risk of RP [23]. The linear regression analysis in this study found that CLD was an influencing factor and an independent influencing factor of V_lung_ 20 for left breast cancer. Therefore, for the plan design of left-side breast cancer combined with clinical guidance, V_lung_ 20 is used as the primary objective function to limit the dose and generate the prediction formula. V_lung_ 20 = 21.48 + 2.8CLD-0.018V_heart_. V_lung_ 20 is positively correlated with CLD and V_heart_. The greater the patient’s CLD, the greater the V_lung_ 20. The measurement of CLD can predict the value of V_lung_ 20 in the ipsilateral lung of left-side breast cancer. Multivariate regression analysis showed that V_PTV_ was not an influencing factor of each limit index of the lung, but it was an influencing factor of D_mean_. This study shows that the total volume of the two lungs and the volume of the affected side lung are not related to the dose volume of each gradient of the affected side lung and the CLD. Univariate linear regression analysis of the V_PTV_ and cardiac indicators of 96 patients with left breast cancer after breast-conserving radiotherapy showed that V_PTV_ is an influencing factor of V_heart_ 30 in patients with left-side breast cancer. Multiple linear regression analysis showed that V_PTV_ is not an independent influencing factor of V_heart_ 30_._ Studies have shown that if the breast volume is greater than 750, V_PTV_ will have a significant effect on the dose volume of the heart and lungs when the tangent field is irradiated in the supine position [24, 25]. This study only observed a weak correlation between V_PTV_ and V_lung_ and did not find any correlation between V_PTV_ and other indicators, which may be related to the relatively small breast volume of the enrolled patients. A retrospective analysis of breast cancer plans using the same irradiation technology by Johnsen et al. [26] showed that the cardiac irradiation dose and volume are greatly affected by the individual anatomy, and approximately 10% of patients experience a cardiac overdose. In this study, under the premise of meeting the clinical requirements, we appropriately reduced the CI value of the target area and strictly controlled the dose volume of the heart and lungs. No overdose of the OAR dose volume was noted.

As a study which explores the influence of individual patient factors on IMRT, it still has some limitations, including a single institution, a small sample size, unevaluated affect factors and a lack of available information on clinical specimens. Besides, the CT scan was performed under free breathing in our study, there are actually techniques that allow to improve the dosimetric calculations of IMRT reducing the interplay effect as well as the protection of organs at risk such as the breath-hold technique. What is more, we will continue to focus on and improve these limitations in the future.

## Conclusion

MHD, CTR, and CLD were identified as factors influencing the results of left breast cancer IMRT. A formula that included the influencing factors MHD, CTR, and CLD can predict the results of the left breast cancer IMRT treatment plan. However, due to the limited number of cases, it is necessary to add more cases to test its reliability. When the result calculated based on the predictive factor deviates too much from the target, the treatment position and technique need to be carefully selected in combination with guidance from clinicians.

## Data Availability

To request the data from this study should contact the corresponding author (meilijindou@126.com).
